# Artificial chromosome technology and its potential application in plants

**DOI:** 10.3389/fpls.2022.970943

**Published:** 2022-09-15

**Authors:** Manman Kan, Tengbo Huang, Panpan Zhao

**Affiliations:** ^1^Guangdong Provincial Key Laboratory for Plant Epigenetics, College of Life Sciences and Oceanography, Shenzhen University, Shenzhen, Guangdong, China; ^2^Key Laboratory of Optoelectronic Devices and Systems of Ministry of Education and Guangdong, College of Physics and Optoelectronic Engineering, Shenzhen University, Shenzhen, China

**Keywords:** plant artificial chromosome, top-down, bottom-up, factors on PACs formation, problems and potential solutions of PACs, exogenous gene(s) integration

## Abstract

Plant genetic engineering and transgenic technology are powerful ways to study the function of genes and improve crop yield and quality in the past few years. However, only a few genes could be transformed by most available genetic engineering and transgenic technologies, so changes still need to be made to meet the demands for high throughput studies, such as investigating the whole genetic pathway of crop traits and avoiding undesirable genes simultaneously in the next generation. Plant artificial chromosome (PAC) technology provides a carrier which allows us to assemble multiple and specific genes to produce a variety of products by minichromosome. However, PAC technology also have limitations that may hinder its further development and application. In this review, we will introduce the current state of PACs technology from PACs formation, factors on PACs formation, problems and potential solutions of PACs and exogenous gene(s) integration.

## Introduction

Gene engineering is termed as recombinant DNA technology and usually used to introduce gene(s) or fragment(s) of interest to organisms to obtain transgenic lines. Genetic engineering technology generally infects plant through agrobacterium infection, protoplast transformation or gene gun, which has greatly promoted the development of world agriculture, such as the application of Bt (*Bacillus thuringiensis*) insect resistance and herbicide resistance. However, the current usage of genetic engineering is limited to the modification of a few genes, and the global application of GM (Genetically Modified) crops still faces challenges, such as their safety and efficacy (Raman, 2017). To meet the demands of people, PAC was developed. PACs, which can stack multiple genes, function as normal chromosomes but do not pair with native chromosomes and have no negative effects to plants (Yu et al., 2016). Compared to the traditional genetic transformation, genetic engineering with PACs has the following advantages: first, PAC can integrate, combine, and express multiple foreign genes which can be either simple stacks of multiple genes or engineering modifications of gene complexes; second, specific site recombination with PACs can integrate exogenous gene(s) in a certain direction without gene expression alteration, gene silencing, and mutation of endogenous gene(s) resulted from random insertion; third, PACs can avoid undesired traits due to genetic linkage in the next generation (Birchler et al., 2016; Yu et al., 2016; Birchler and Swyers, 2020). Therefore, PAC is promised to promote the green revolution of genetic engineering technology and become an effective approach for crop improvement. In this review, we will introduce the current state of the plant artificial chromosome technology.

## Plant artificial chromosomes

Artificial chromosome is a powerful research tool in both genome and gene function studies in recent years, especially in mammalian and yeast (Kouprina et al., 2018; Pesenti et al., 2018; Shao et al., 2018; Moralli and Monaco, 2020). There are two approaches to construct artificial chromosome: top-down and bottom-up (Kazuki and Oshimura, 2011). Top-down is based on telomere-mediated chromosome truncation (TMCT), which seeds a new telomere at DNA integration site and generates truncated chromosome by introducing telomeric sequence into plant. Bottom-up, also known as *de novo* construction of chromosome, is usually assembled *in vitro* with autonomously replicating sequence (ARS), centromere and/or telomere. In addition to the essential elements, selection markers and a site-specific recombination system, in which target genes could be inserted, are also needed.

**Figure 1:**
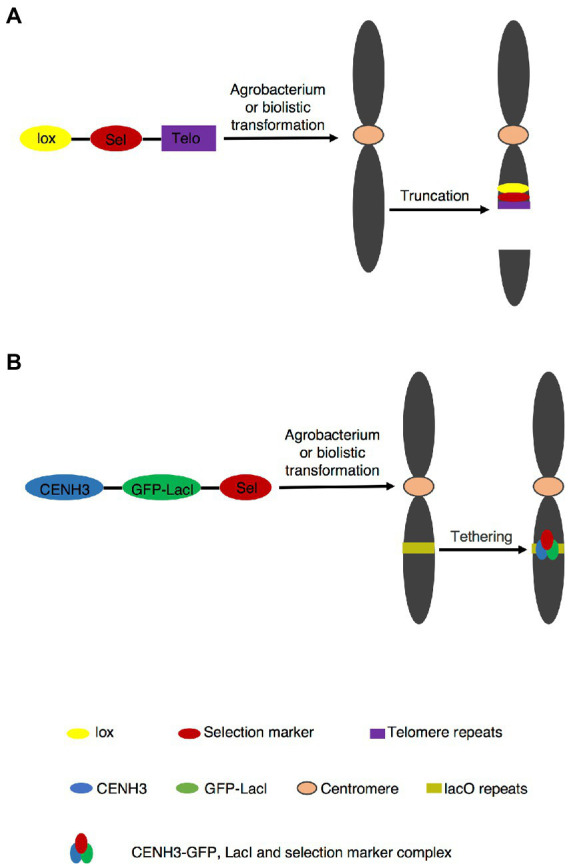
PACs generation by telomere-mediated chromosome truncation and *de novo* centromere formation. **(A)** Telomere mediated chromosome truncation *via* agrobacterium transformation or biotransformation. The site of truncation is random. The truncated fragment without centromere part is lost in subsequent cell divisions. **(B)** *De novo* centromere formation. Targeting CENH3-GFP-LacI to plant containing lacO tandem repeat arrays to form centromere.

Telomere is a special nucleoprotein complex assembled on the ends of chromosomes, which contains short DNA repeats and telomerase to maintain chromosomes stability. Most higher plants share the Arabidopsis (*Arabidopsis thaliana*)-type telomere repeat TTTAGGG, whereas a few species have a different telomere repeat sequence. For example, the core sequence of telomere repeats in Asparagales and Solanaceae are TTAGGG and TTTTTTAGGG, respectively (Peska et al., 2015). *De novo* synthesized telomeric fragment was inserted and seeded a new telomere at the insertion site (Yin et al., 2021). Subsequently, artificial chromosome is generated by truncating original chromosome (Figure 1A). TMCT in plants was first reported in 2006 by transferring 2.6 kb Arabidopsis-type telomere repeat into maize (*Zea mays*) through agrobacterium-mediated transformation (Yu et al., 2006). Both A and B chromosomes in maize were truncated. For the A chromosome, TMCT occurred more frequently at long arm than at short arm (Yu et al., 2006). Moreover, the TMCT was faithfully transmitted to the next generation, and truncated chromosome can recombine with normal chromosome *via* Cre/lox or FLP/FRT recombination system, indicating the successful application of the TMCT platform (Yu et al., 2007; Xu et al., 2012). Diverse length of Arabidopsis-type telomere repeat TTTAGGG were inserted into maize (Yu et al., 2006, 2007; Gaeta et al., 2011; Swyers et al., 2016), rice (*Oryza sativa*; Xu et al., 2012; Xu and Yu, 2016), Arabidopsis (Nelson et al., 2011; Teo et al., 2011), barley (*Hordeum vulgare*; Kapusi et al., 2012), wheat (*Triticum aestivum*; Yuan et al., 2017), and *Brassica napus* (Yan et al., 2017; Yin et al., 2021) by agrobacterium-mediated transformation or biotransformation, and TMCT were generated, which revealed that minichromosomes could be created in most of plant species by TMCT approach.

Unlike telomere-mediated chromosome truncation, *de novo* construction of chromosome assembles not only telomere, but also ARS and centromere elements *in vitro* into a linear or circular plasmid as a complete candidate functional chromosome, which is transformed into cells rather than derived from a native chromosome. Centromere is the essential element for *de novo* construction of a chromosome. However, centromeres containing all functional DNA elements may be inactive (Han et al., 2006). In addition, reactivation and *de novo* centromere formation could happen on fragments derived from the progenitor possessing an inactive centromere (Douglas et al., 2021). These facts indicate formation of a functional centromere does not rely solely on its DNA sequences (Han et al., 2006). Carlson et al. (2007) constructed a circular plasmid carrying 19 kb centromere and delivered it into embryogenic maize tissues. They finally generated one *de novo* chromosome, but this chromosome was not stable in next generation (Carlson et al., 2007).This might be due to modifications of the inserted centromere or epigenetic effects *in planta.* (Song et al., 2021; Wang et al., 2021). Ananiev et al. (2009) used the core set of centromere, maize 18–26S rDNA containing the replication origin and 239 bp synthetic telomeric sequence to construct minichromosome shuttle vectors. As a result, 7 putative functional *de novo* minichromosomes were generated and confirmed by CENPC antibody (Ananiev et al., 2009). Nevertheless, there are some arguments because these minichromosomes were greatly larger than the original plasmids, indicating the integration of exogenous DNA fragments into *de novo* chromosomes (Yu et al., 2016).

## Factors on the formation of PAC

To date, minichromosomes have been produced in some plant species while the efficiency is quite low. There are some parameters affecting PAC formation. Centromeres contain several unique sequences, including centromere-specific satellite repeat, CentO, and a centromere-specific retrotransposon (Naish et al., 2021; Song et al., 2021; Wang et al., 2021), and the functionality of a centromere is influenced by the epigenetic status of the centromere protein (CENPA/CENH3; Gascoigne and Cheeseman, 2011; Wong et al., 2020). However, how the function of centromere is activated remains largely underexplored because of the highly repetitive sequence and complex structure of centromere. Recently, centromere analysis basing CENH3 chromatin immunoprecipitation and sequencing or gap-free genome assembly revealed some details, such as, epigenetic movement of the centromere to slightly or drastically different positions (Walkowiak et al., 2020)， the greatest higher-order repetition of satellite repeat showing the highest CENH3 occupancy and CG methylation (Naish et al., 2021), different CENH3 deposition sites in the same *Cent*O on different centromeres (Song et al., 2021) and the invasion of *ATHILA* retrotransposons leading to disruption of centromeres’ epigenetic organization (Naish et al., 2021). Also, due to the effects of epigenetic modifications on centromeres, *de no* synthesized centromeres directly transformed into plant cell were almost inactive (Carlson et al., 2007; Ananiev et al., 2009).

The internal telomere originates from recombination, ancestral chromosome fusions, or double-strand break (DSB) repairs (Azzalin et al., 2001; Nergadze et al., 2004; Liu et al., 2021). Chromosomal DSBs can be repaired by homology directed repair (HDR) and nonhomologous end-joining (NHEJ), which is the major chromosome-repair mechanism to join DSBs to a fusion product in plants and animals (Nergadze et al., 2004; de Lange, 2009). DSBs repair related proteins and DNA checkpoint proteins are closely associated with telomeres, and are essential for telomere maintenance (Nergadze et al., 2004). For example, DNA checkpoint protein MRT2 and DSBs repair complex Mre11/Rad50/Nbs1 and Mre11/Rad50/Xrs2, are required for telomere maintenance in yeast and humans (Diede and Gottschling, 2001; Larrivee et al., 2004; Chai et al., 2006). In addition, TRF2 binds to the TTAGGG sequences in double-stranded DNA to repress DNA repair pathways that could harm telomeres (de Lange, 2009). TRF2 loss from telomeres renders them highly susceptible to Ku70/80 and DNA ligase IV (lig4)-dependent classical-NHEJ and resulted in end-to-end chromosome fusions (Ruis et al., 2021). Ku, a core component of NHEJ, promotes telomerase and a heterochromatin factor, Sir4 recruitment at DSBs and protects natural telomeres from chromosome fusion in yeast (Marcand, 2014; Zahid et al., 2021). In Arabidopsis, loss function of Ku70 exhibited dramatically lower TMCT frequency compared to wild-type (Nelson et al., 2011) indicating the special role of Ku in telomere formation. LIG4 is an ATP-dependent DNA ligase dedicated to NHEJ (Palmbos et al., 2008; Chiruvella et al., 2013). *lig4* transformants showed a significant decrease of TMCT relative to wild-type plant (Nelson et al., 2011). Together with these results, proteins shared between DSB repairs and telomere maintenance make the sense that DSB repairs make chromosomal truncation more efficient.

The presence of telomeric sequence seeds telomere frequently, though it is not a prerequisite for the generation of a telomere (Yu et al., 2006; Vega et al., 2008; Nelson et al., 2011; Teo et al., 2011; Schrumpfova and Fajkus, 2020). Furthermore, longer telomeric sequence seems more easily to form truncated chromosome in humans and plants (Table 1; Farr et al., 1991; Barnett et al., 1993; Nelson et al., 2011). To date, the minimum telomeric sequence to establish new telomere was 100 bp with 25% *de novo* telomere formation (DNTF) frequency, while 900 bp telomeric sequence insertion increased the DNTF frequency to 55% in Arabidopsis (Nelson et al., 2011). However, long fragment in agrobacterium is unstable and telomeric sequence inhibited transformation (Nelson et al., 2011; Teo et al., 2011). Therefore, the balance between the length of telomeric sequence and transformation efficiency is crucial for chromosome truncation in different plant species.

**Table 1:** TMCT frequency resulted from divers length telomeric sequence integration.

Species	Polidy	Telomere sequence size (bp)	Efficiency (%)	Reference
Arabidopsis	2n	2,600	10	Nelson et al. (2011)
Arabidopsis	4n	100	25	Nelson et al. (2011)
Arabidopsis	4n	900	55	Nelson et al. (2011)
Arabidopsis	4n	2,600	58	Nelson et al. (2011)
Human	2n	500	22	Farr et al. (1991)
Human	2n	1,000	55	Barnett et al. (1993)

Moreover, the ploidy of host plant also affects TMCT. In the same species, polyploid showed more transgenes than diploid (Christiansen et al., 2005; Teo et al., 2011), which indicates more DBS repair events will happen and subsequently increase TMCT frequency. In addition to more transgene events, polyploid also exhibited higher TMCT frequency than diploid. TMCT frequency in tetraploid Arabidopsis is 56%, which is much higher than that of diploid Arabidopsis (10%) under the same number of T-DNA insertions (Nelson et al., 2011). Moreover, when TMCT occurred in tetraploid and diploid barley, chromosome numbers in all diploid materials were increased to tetraploid during tissue culture (Kapusi et al., 2012), indicating that truncated chromosomes can be present only in tetraploid background, which compensates the loss of the respective chromosome region by TMCT (Kapusi et al., 2012).

## Problems of PAC application in plants and potential solutions

Top-down and bottom-up approaches have already been applied in yeast and mammalian cells (Murray and Szostak, 1983; Harrington et al., 1997; Ikeno et al., 1998; Henning et al., 1999; Ebersole et al., 2000; Grimes et al., 2001; Logsdon et al., 2019; Kouprina et al., 2020). In plants, the application of PACs is rarely seen, which is attributable to a number of potential problems. Bottom-up approach seems less feasible because of the strong epigenetic influence of CENPA/CENH3 on centromere, which is functional in separation of sister chromatids at anaphase I (Gascoigne and Cheeseman, 2011; Gent et al., 2012; Birchler et al., 2016). Therefore, simply cloning plant centromere sequences and re-introduction them into plant cells failed to form active centromeres (Phan et al., 2007). *De novo* construction of human artificial chromosome has been established by targeting CENPs-tetR or LacI fusion protein to tetO or lacO tandem repeat arrays, in which active centromeres were generated (Kouprina et al., 2018; Pesenti et al., 2018; Logsdon et al., 2019; Ohzeki et al., 2020; Okazaki et al., 2022). In addition, *de novo* active centromere was generated by targeting CENH3-GFP-LacI to lacO tandem repeat arrays in Arabidopsis (Figure 1B; Teo et al., 2013). Consulting these strategies of human artificial chromosome may provide the possibility to successfully construct plant *de novo* chromosome in the future.

Construction of minichromosome by TMCT has been successfully used in plants (Yu et al., 2006, 2007; Nelson et al., 2011; Teo et al., 2011; Kapusi et al., 2012; Xu et al., 2012; Yuan et al., 2017; Yin et al., 2021). However, TMCT also confronted many problems. When normal chromosomes are truncated, the loss of chromosome regions may be detrimental to plant growth and viability, resulting in abnormal plant growth or death (Yin et al., 2021). One of the solutions is based on truncating redundant chromosomes, such as the B chromosome in maize (Yu et al., 2006, 2007; Gaeta et al., 2013), but not all plant species contain redundant chromosomes. Another way is to use polyploid as the starting material, which will recompense the lost region derived from chromosome truncation (Nelson et al., 2011; Kapusi et al., 2012). In previous studies, TMCT derived from T-DNA insertion occurred randomly on the chromosome, resulting in sterility or death of plants (Yu et al., 2006; Nelson et al., 2011; Teo et al., 2011; Xu et al., 2012). Therefore, precisely modifying genome sequence is extremely important. Homologous recombination is one of the best methods to ensure precise modification and have been applied in plants (Terada et al., 2002). However, the efficiency of homologous recombination is quite low, and genome targeting highly relies on DSBs at recognized sites by engineered nucleases, such as zinc finger nucleases (ZFNs), transcriptional activator-like effector nucleases (TALENs), and clustered regularly spaced short palindromic repeats (CRISPR)/Cas9 systems (Gupta et al., 2019; Janik et al., 2020). Studies also revealed that mixing two or three plasmids and transforming them into plant cells by biolistic transformation resulted in efficient chromosomal truncation (Xu et al., 2012; Gaeta et al., 2013). The gene target construct co-transformed with the telomeric sequence containing construct would generate minichromosome at certain site to reduce the disadvantages resulted from random chromosome truncation.

The top-down and bottom-up approaches also face some common problems. Previous studies have shown that minichromosomes do not pair correctly and lead to poor transmission efficiency (Han et al., 2007; Masonbrink et al., 2012; Dawe, 2020). Therefore, placing pollen selection genes on minichromosomes and screening plants containing these markers may be the most appropriate way to solve this problem (Birchler et al., 2016). Secondly, large fragment is difficult to be transformed into plant cell by current plant transformation methods, such as agrobacterium mediated transformation and biotransformation (Dawe, 2020). Moreover, minichromosome transferring between different species is quite difficult due to hybridization barrier. To settle the problem, protoplast fusion techniques may be implemented. Studies have shown that the protoplast fusion technology can recombine the chromosomes of two species, such as the fusion of tobacco (*Nicotiana tabacum L.*) and Arabidopsis protoplasts using Cre/lox recombinase system (Kouprina et al., 2020), wheat and maize protoplasts fused by polyethylene-mediated transformation (Szabados et al., 1981). In addition, utilizing *de novo* constructed human chromosome in yeast and protoplast fusion techniques between yeast and human cells, *de novo* constructed chromosome were successfully moved to human cells from yeast (Kouprina et al., 2018; Kim et al., 2021).

## Strategies of exogenous gene(s) integration

PAC could be used as a platform for stacking transgenes (Yan et al., 2017). Therefore, approaches to precisely stack genes in minichromosome is very important. DNA recombinase-mediated site-specific integration (SSI) is a promising technology to place transgenes into a certain site in plant genome (Srivastava and Thomson, 2016; Pathak and Srivastava, 2020).

Several site-specific DNA recombination systems, such as, Cre/lox, FLP/FRT, R/RS and phiC31/att, have been used in SSI studies (Ow, 2002; Li et al., 2009; Elias et al., 2020; Pathak and Srivastava, 2020). In previous studies, by crossing two transgenic plants, one contains promoterless lox77/FRT-reporter gene on a minichromosome or normal chromosome and the other harboring promoter-lox66/FRT-Cre/FLP, the minichromosome and normal chromosome can be recombined (Yu et al., 2007; Xu et al., 2012). These results demonstrated that the Cre/lox and FLP/FRT SSI system can be used for site-specific recombination in a minichromosome. Using Cre/lox or FLP/FRT to transform two oppositely oriented identical recombinase recognition sites containing circular plasmid into a transgenic plant harboring two same recombinase recognition sites resulted in cassette exchange between the donor and a previously placed target (Nanto et al., 2005; Pathak and Srivastava, 2020). This technique is called recombinase-mediated cassette exchange (RMCE). RMCE with two recognition sites provides a flexible way for genome targeting (Ow, 2002; Li et al., 2009). However, two identical recombinase recognition sites lead to excision or flipping of the flanked DNA segment. By crossing the minichromosome containing two oppositely oriented loxP to a plant expressing Cre, the Bar selection gene between two loxP was removed, indicating minichromosomes can be modified *in vivo* as normal chromosomes by site-specific recombinases (Gaeta et al., 2013). Mutation recognition sites and combination of two site-specific recombinases system can be used to prevent the excision and flipping, such as, a loxP and a mutant *loxP511*, *lox5171* or *lox2272* (Trinh and Morrison, 2000; Longhese, 2008; Hacker et al., 2017), a FRT and a mutant *FRT3* (Horn and Handler, 2005), or dual RMCE of Cre/lox and FLP/FRT (Lauth et al., 2002; Osterwalder et al., 2010; Voziyanova et al., 2017; Kim et al., 2019). DNA cassette exchange is reversible in RMCE. When donor cassette contains a third recognition site, this third recognition site can be inserted into the original targeting sites by RMCE for a new round of RMCE to stack genes (Li et al., 2009). If more incompatible recognition sites are available, repeating the process of RMCE would achieve gene stacking in engineered minichromosomes.

## Perspective

Over the past few decades, the amount of arable land on earth has been decreasing as the number of people increase. Genetic engineering technology needs to be improved to an advanced platform to meet the demands of food quantity and quality. PAC has attracted attention for its ability to stack and manipulate genes, which allow us to add engineered complex metabolic pathways to improve plant nutrition or to produce valuable medicines or biofuels, potentially changing the bioeconomy. Despite the difficulties faced of PACs, with the potential solutions and progress of science and technology, improved PAC technology will eventually play an important role in genetic engineering to produce more and higher quality agricultural products to meet future demand.

## Author contributions

MK, TH and PZ were contributed to the writing of this review. All authors contributed to the article and approved it for publication.

## Funding

The studies on plant artificial chromosome in Huang Lab is supported by the National Key R&D Project (Grant No. 2019YFA0903900) from the Ministry of Science and Technology of China and Guangdong Special Support Program for Young Talents in Innovation Research of Science and Technology (2019TQ05N940).

## Conflict of interest

The authors declare that the research was conducted in the absence of any commercial or financial relationships that could be construed as a potential conflict of interest.

## Publisher’s note

All claims expressed in this article are solely those of the authors and do not necessarily represent those of their affiliated organizations, or those of the publisher, the editors and the reviewers. Any product that may be evaluated in this article, or claim that may be made by its manufacturer, is not guaranteed or endorsed by the publisher.
